# Dual-Role Ubiquitination Regulation Shuttling the Entire Life Cycle of the Flaviviridae

**DOI:** 10.3389/fmicb.2022.835344

**Published:** 2022-05-06

**Authors:** Dongjie Cai, Lingli Liu, Bin Tian, Xingxin Fu, Qiyuan Yang, Jie Chen, Yilin Zhang, Jing Fang, Liuhong Shen, Ya Wang, Liping Gou, Zhicai Zuo

**Affiliations:** ^1^Key Laboratory of Animal Disease and Human Health of Sichuan Province, College of Veterinary Medicine, Sichuan Agricultural University, Chengdu, China; ^2^Institute of Preventive Veterinary Medicine, Sichuan Agricultural University, Chengdu, China; ^3^Laboratory of Animal Disease Prevention and Control Center, Agriculture and Rural Affairs Bureau of Luoping County, Luoping, China; ^4^Department of Basic Veterinary Medicine, Sichuan Agricultural University, Chengdu, China

**Keywords:** ubiquitin system, antiviral immunity, Flaviviridae, HCV, CSFV, TRIM proteins

## Abstract

Ubiquitination is a reversible protein post-translational modification that regulates various pivotal physiological and pathological processes in all eukaryotes. Recently, the antiviral immune response is enhanced by the regulation of ubiquitination. Intriguingly, Flaviviridae viruses can ingeniously hijack the ubiquitination system to help them survive, which has become a hot topic among worldwide researchers. The Flaviviridae family members, such as HCV and CSFV, can cause serious diseases of humans and animals around the world. The multiple roles of ubiquitination involved in the life cycle of Flaviviridae family would open new sight for future development of antiviral tactic. Here, we discuss recent advances with regard to functional roles of ubiquitination and some ubiquitin-like modifications in the life cycle of Flaviviridae infection, shedding new light on the antiviral mechanism research and therapeutic drug development.

## Introduction

Ubiquitination is one of the means of post-translational modifications (PTMs) that are previously considered to coordinate the protein activities in cells. Ubiquitination modification system take part in many different cellular processes: An example is protein degradation (Kwon and Ciechanover, 2017), endocytosis (Mukhopadhyay and Riezman, 2007), DNA repair (Bergink and Jentsch, 2009), inflammation (Bianchi and Meier, 2009), autophagy (Kirkin et al., 2009), immunity (Hu and Sun, 2016), and apoptosis (Vucic et al., 2011). However, some remarkable recent advances in the ubiquitin field proposed that ubiquitination- and some ubiquitin-like-modification can be utilized by viruses to facilitate their own replication (Luo, 2016; Rudnicka and Yamauchi, 2016; Sun et al., 2019; Gu and Jan Fada, 2020; Valerdi et al., 2021). In particular, the ubiquitination and some ubiquitin-like modifications of the Flaviviridae virus proteins play key roles in promoting virus enter cell (Dejarnac et al., 2018; Giraldo et al., 2020b), releasing viral RNA during internalization (Byk et al., 2016; Wang et al., 2016b; Ramanathan et al., 2020), assisting virus conquer the host antiviral immunity (Lee et al., 2014; Kwak et al., 2016; Su et al., 2016; Abe et al., 2020; Liang et al., 2021), and accelerating virus assembly, transportation, budding and egress (Ko et al., 2010; Kumar et al., 2019). These reports indicate that Flaviviridae viruses can ingeniously use ubiquitination and some ubiquitin-like modifications to optimizing their infection at each step of their life cycle. Now, how Flaviviridae viruses seize the ubiquitination system to climate their infection has caught the eye of several researchers working in the field.

This review will debate the roles of ubiquitination and some ubiquitin-like modifications in the life cycle of Flaviviridae family by dissecting the process into several processes: (1) Attachment and entry; (2) Internalization; (3) Replication; and (4) Assembly and Release. Understanding how viruses make use of the ubiquitination can pave new ways to develop antiviral strategies, which may help us to identify common basic host-dependent ubiquitin factors. These factors will be suitable for the development of broad-spectrum antivirals with pan-flaviviral activity.

## The Ubiquitin System

**Figure 1:**
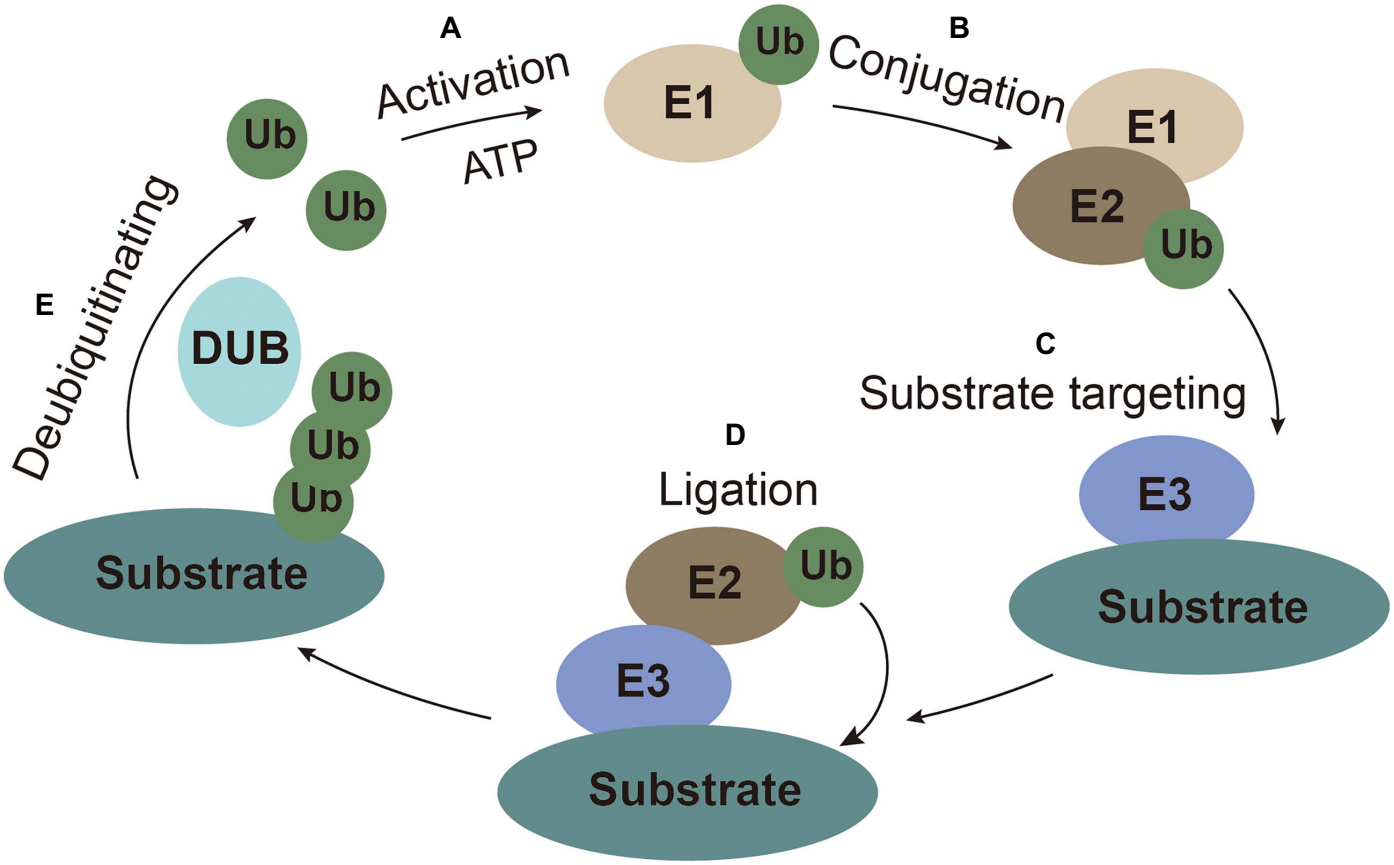
The ubiquitin system. **(A)** Activation: ubiquitin is activated and bound to E1 with ATP as an energy source. **(B)** Conjugation: ubiquitin is conjugated to E2. **(C)** Substrate targeting: substrates are targeted by E3. **(D)** Ligation: ubiquitin is ligated to the substrate. **(E)** Deubiquitinating: ubiquitin is recycled by the deubiquitinating enzymes.

Ubiquitin (Ub), a highly conserved small peptide, is a compact spherical structure composed of 76 amino acids and has an exposed C-terminal tail that can be covalently linked to substrate proteins (Husnjak and Dikic, 2012). The 7 lysines (K6, K11, K27, K29, K33, K48, and K63) and N-terminus of Ub can be combined with another Ub to form polyubiquitin chains on the substrates to perform corresponding special regulatory functions (Kirisako et al., 2006; Xu et al., 2009). Ubiquitination modification is achieved through a series of enzymatic reactions carried out by E1 (ubiquitin-activating enzymes), E2 (ubiquitin-conjugating enzyme), and E3 (ubiquitin ligases) coordinately. The E1 enzyme activates ubiquitin in an ATP-dependent manner. Ub molecule is then transferred to the ubiquitin-conjugating enzyme E2 (Ye and Rape, 2009). The ubiquitin-ligase E3 recognizes E2 and substrate, and then transfers the ubiquitin from E2 to substrate for ubiquitination (Gao and Luo, 2006). Ubiquitination is a reversible process, termed as deubiquitination, which is performed by deubiquitinating enzymes (DUBs). DUBs hydrolyze and recycle polyubiquitin chains from the substrates (Popovic et al., 2014; Figure 1).

Different ubiquitin linkages on the substrates usually have particular cellular functions. The K48-linked polyubiquitin chain, the most common ubiquitin linkage, account for more than 50% of all linkages, which triggers proteasome-dependent substrate degradation (Thrower et al., 2000). The second most common form is the K63 polyubiquitin chain, which mainly participate in non-proteolytic signaling, including protein transport, DNA repair, autophagy, and inflammation (Jacobson et al., 2009).

## The Molecular Structure of Flaviviridae Viruses

**Figure 2:**
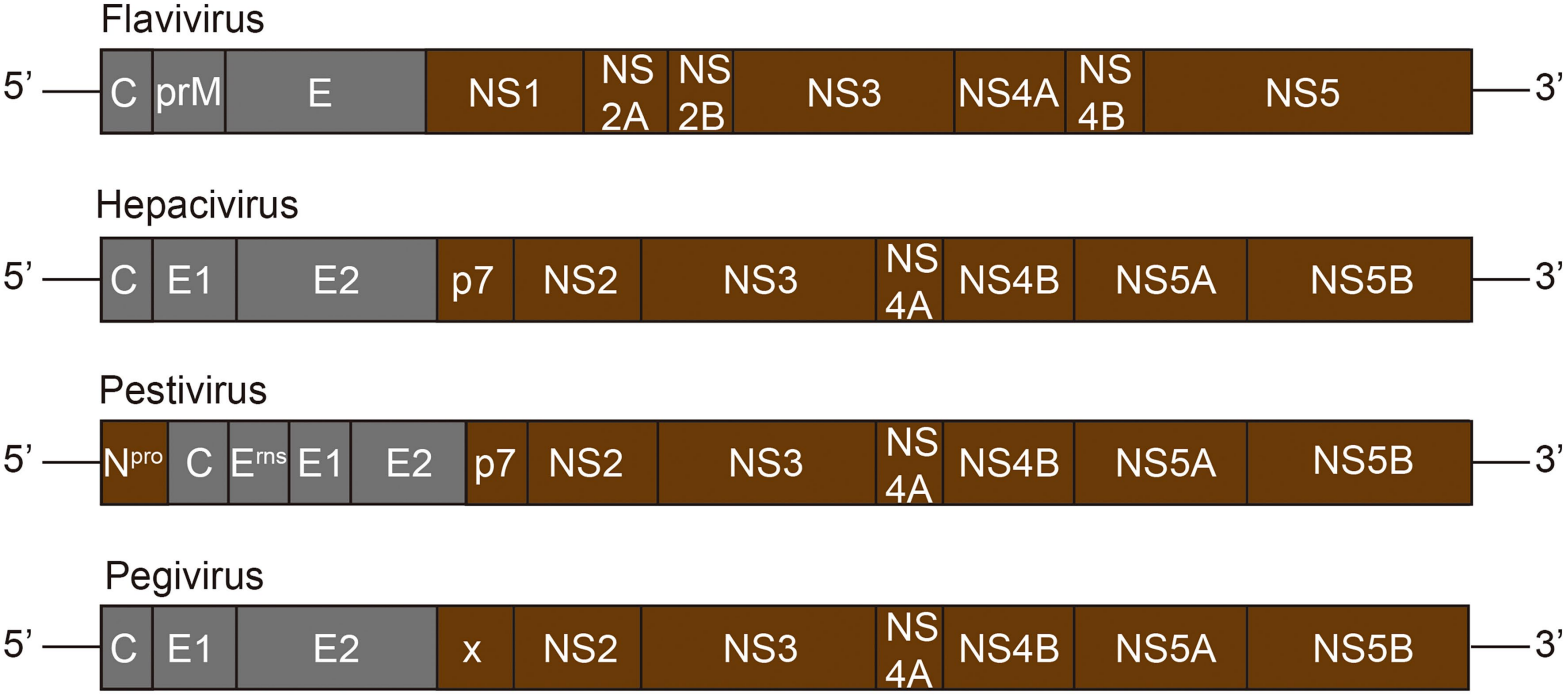
Genome structure of Flaviviridae family. The Flaviviridae genome is composed of a 5′-terminal non-coding region (NCR) and a 3′-terminal NCR, and a single long ORF (open reading frame) encoding the polyprotein. The polyprotein is cleaved by viral and host proteases to produce about 10–12 mature proteins. Grey represents viral structural proteins, while brown represents non-structural proteins.

The Flaviviridae family consists of four genera: *Flavivirus*, *Hepacivirus*, *Pestivirus*, and *Pegivirus* (Stapleton et al., 2011). The Flaviviridae virus infection has a great impact on human and animal (Neufeldt et al., 2018). The virion surface of the Flviviridae is symmetrical icosahedral, and the outermost layer is coated with glycoprotein consisting of repetitive units of envelope protein E, which can be inserted into lipid membrane of cells (Kuhn et al., 2002). The inner core of Flaviviridae virus is a single positive-stranded RNA molecule combined with multiple copies of capsid protein E (Barrows et al., 2018). The Flaviviridae family genome structure is illustrated in Figure 2. The length of their genome is about 9.6–12.3 kb, composing by a large open reading frame (ORF) with an untranslated region (UTR) at both ends (Zhang Z. et al., 2019).

The virions are composed with structural proteins, including a capsid protein E, the prM protein, and two to three copies of envelope proteins E (Neufeldt et al., 2018). The non-structural proteins are mainly involved in the viral replication and assembly, and interaction with cellular proteins to optimize the infectivity and modulate the immunity of the host. NS1 protein, unique to the *Flavivirus* genus, is the only non-structural protein present in the endoplasmic reticulum (Neufeldt et al., 2018). The NS1 and NS2A of Flaviviruses are required for RNA replication and production of infectious particles. The P7 and NS2 of the *Hepacivirus* virus support the assembly and release of virions, and the C-terminal domain of NS2 contains cysteine proteases that catalyze NS2-NS3 junction cleavage (Neufeldt et al., 2018). NS2B of *Flavivirus* or NS4A of *Hepacivirus* acts as a cofactor of NS3 protease and recruits NS3 to the ER membrane. NS3, a multifunctional protein, is necessary for RNA replication with the activity of serine protease, RNA helicase, and nucleoside triphosphatase (NTPase; Dubuisson and Cosset, 2014). The NS4A of *Flavivirus* is an integral membrane protein engaged in membrane rearrangement to form a viral replication complex, while the NS4B of *Hepacivirus* virus plays this role in this process (Neufeldt et al., 2018). Both NS5 of *Flavivirus* and NS5B of *Hepacivirus* virus have the RNA-dependent RNA polymerase activity, which is responsible for viral RNA synthesis (Moradpour and Penin, 2013). In addition, NS5A of the *Hepacivirus* virus is a phosphoprotein involved in interaction with host factors and RNA binding (Neufeldt et al., 2018).

## The Role of Ubiquitination in the Life Cycle of Flaviviridae

**Figure 3:**
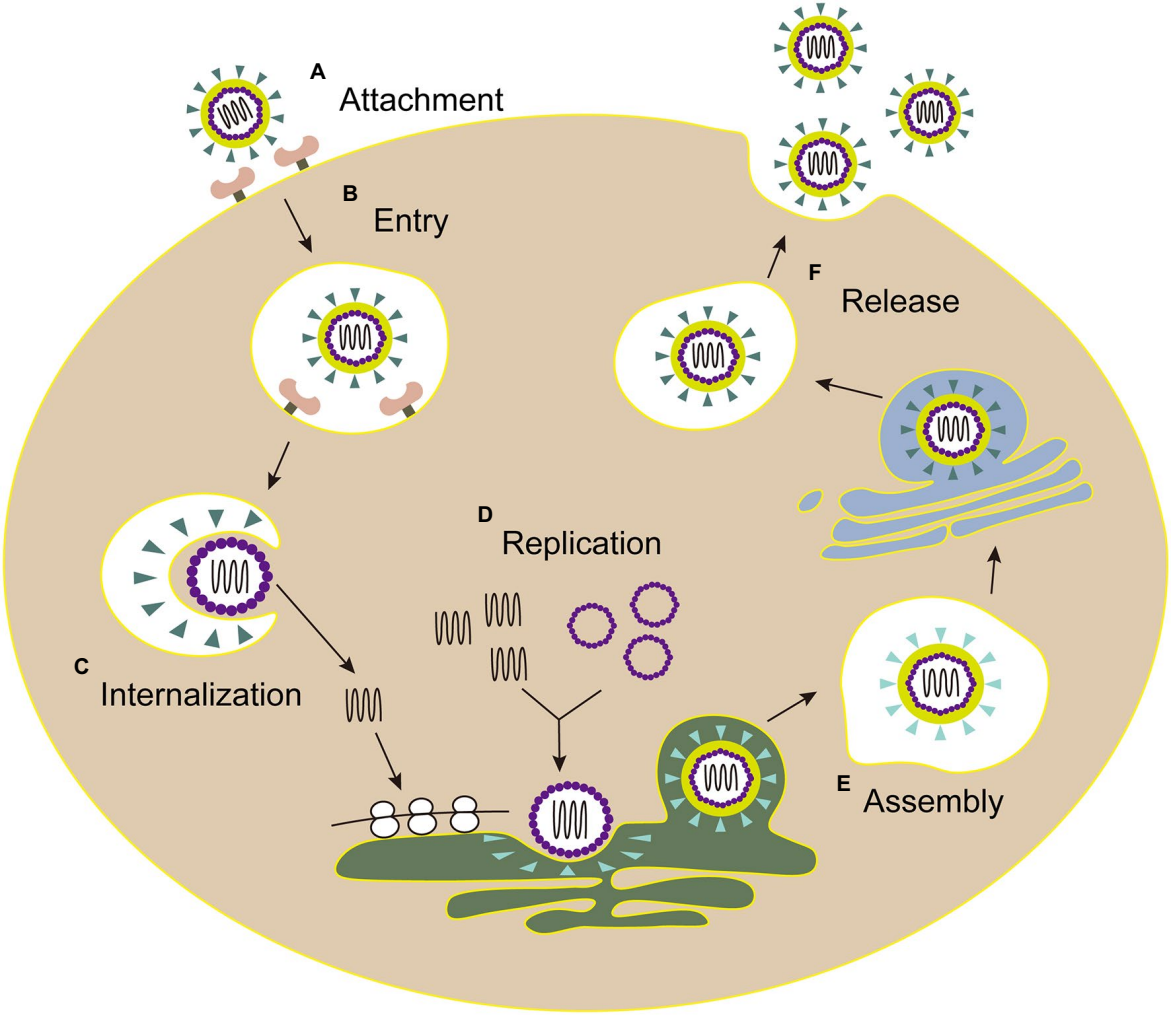
The life cycle of Flaviviridae. **(A)** Attachment: E envelope protein of viral particles interacts with the cell receptor. **(B)** Entry: virus enters the host cell by clathrin-mediated endocytosis. **(C)** Internalization: the low pH environment in the endosome triggers a conformational change of the E glycoprotein, and then the viral envelope fuses with the endosomal membrane. Following the nucleocapsid of virus is released into the cytoplasm. **(D)** Replication: the genome is transported into cytoplasm, then the viral RNA and proteins are synthesized in the endoplasmic reticulum. **(E)** Assembly: the viral particle is assembled in the adjacent endoplasmic reticulum, then immature virus particles egress (blue triangle, envelope proteins). **(F)** Release: mature virions (green triangle, envelope proteins) are released after the Golgi apparatus modification.

The life cycles of Flaviviridae viruses are highly similar, including attachment, entry, internalization, replication, assembly, and release (Figure 3).

### Ubiquitination Assists With Attachment and Entry for Flaviviridae Virus

Flaviviridae viruses attach the target cells *via* the interaction between the E protein and its cognitive receptor (Perera-Lecoin et al., 2013), and then entry into cells through endocytosis in a clathrin-mediated way.

**Figure 4:**
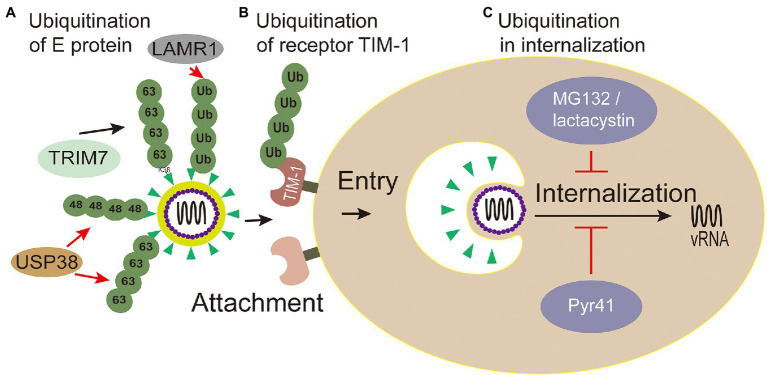
Ubiquitin in viral attachment, entry and internalization. **(A)** Ubiquitination of E protein: ubiquitination of TRIM7-mediated K63-linked on the K38 residue of the E protein promotes the effective adhesion of ZIKV to host receptor; USP38 binds to ZIKV envelope (E) protein and attenuates K48-linked and K63-linked polyubiquitination; LAMR1 binds with E protein to attenuate the ubiquitination of E protein. **(B)** Ubiquitination of receptor TIM-1: the ubiquitination of TIM-1 promotes the endocytosis of DENV. **(C)** Ubiquitination in virus internalization: MG132 and lactacystin (proteasome inhibitors) reduced the productive virions internalization of Japanese encephalitis virus (JEV). The action of Pyr41 (E1 inhibitor) confirmed that ubiquitination is necessary for the uncoating process of DENV and YFV.

E envelope protein plays a major role in the processes of attaching host cells and membrane fusion (Pierson and Kielian, 2013). Co-immunoprecipitation assays with Huh7 cells infected with ZIKV confirmed that E of ZIKV was K63-linked ubiquitinated (Giraldo et al., 2020b). Further research shows that there are two ubiquitination sites (residues K38 and K281) on E of ZIKV. Yet, the results showed only the replication level of ZIKV E (K38R)-mutant, but not E (K281R)-mutant, significantly reduced in testis (15P-1) and liver (Huh7) cells (Giraldo et al., 2020b), which indicates that the residue K38 of E protein is the key site for ubiquitination. Also, the residue K38 of E protein is conserved among *Flavivirus* (Dai et al., 2016), whereas residue K281 is not (Kostyuchenko et al., 2016). Furthermore, the specific ubiquitination on the residue K38 of the E protein was identified in a TRIM7-mediated K63-linked way to promote the effective adhesion of the virus to the host receptor, which is the reason for effective attachment of virus to host cells and at least partially mediated by the TIM-1 receptor (Giraldo et al., 2020b; Figure 4). Some researchers made wild-type and mutant ZIKV labeled with lipophilic dye (DiOC18) to analyze endosome-virus membrane fusion. As a result, compared with wild-type ZIKV in JEG-3 and A549 cells, the ability of E(K38R)-mutant ZIKV promoting virus-endosome fusion was significantly reduced. This report illustrated that the ubiquitination of E is essential for ZIKV entry, and the anti-K63-linked ubiquitin antibody has neutralizing activity against ZIKV *in vivo* (Giraldo et al., 2020b), which suggests that the ubiquitination of K63 may provide a novel way to treat ZIKV.

Meanwhile, inhibiting Zika virus infection by removing envelope protein ubiquitination mediated by the host cells has also been reported. Ubiquitin-specific peptidase 38 (USP38), a member of the ubiquitin specific processing enzyme family, has been reported to inhibit type I interferon signaling during viral infection (Lin et al., 2016). It has been reported that USP38 addresses inflammation by binding histone ubiquitination and methylation (Zhao et al., 2020). Wang et al. (2021) reported that USP38 binds to ZIKV envelope (E) protein through its C-terminal domain and attenuates polyubiquitination of its K48 and K63 linkage, thus inhibiting ZIKV infection. These reports demonstrate that USP38 may be a potential therapeutic target for the treatment and prevention of ZIKV infection. Laminin receptor 1 (LAMR1) is a ubiquitously multifaceted protein. LAMR1 can bind to E protein of ZIKV through its intracellular region, and attenuate E protein ubiquitination by recruiting di-ubiquitin eukaryotic translation initiation factor 3 subunit 5 (EIF3S5; Hu et al., 2021; Figure 4).

TIM-1 is the main receptor that mediates Dengue Virus (DENV) infectious virions entry into cells (Meertens et al., 2012; Hamel et al., 2015). TIM-1 knockout A549, Huh7.5, and 769P cells by CRISPR/Cas9 were generated, and the infectivity of DENV was significantly inhibited in these cells with TIM-1 deficiency (Dejarnac et al., 2018). Moreover, the amount of internalized DENV RNA was significantly reduced in TIM-1 KO cells (Dejarnac et al., 2018). Further tests showed that TIM-1 is ubiquitinated on two lysine residues K338 and K346 of its cytoplasmic domain. The HeLa cells expressing TIM-1 cytoplasmic tail mutants, in which one or two lysine residues at positions 338 and 346 were replaced by arginine (K338R, K346R, and KKRR), internalized fewer virions than wild type cells, indicating that ubiquitination in TIM-1 played a key role in the endocytosis of DENV (Dejarnac et al., 2018; Figure 4).

To sum up, the Flaviviridae virus decorate ubiquitination on its protein E and host receptor TIM-1 that are critical for its attachment and entry. Attachment and entry are the crucial steps for Flaviviridae viral infection, and the ubiquitination of E and TIM-1 can be a hint for the inhibitor development against multiple viruses.

### Ubiquitination Involved With Flaviviridae Virus Internalization

After virus entering the cell through receptor-mediated endocytosis, internalization is followed. Internalization is the process that internalized virions are released from endosomes (Heinz et al., 1994; Stiasny et al., 2011). In detail, the virus envelop does not fuse with endosome membrane until the endosome matures, following uncoating of the nucleocapsid, then releasing viral RNA to the cytoplasm (Heinz et al., 1994). Although the basic mechanisms for Flaviviridae entry and fusion are well understood, there is little known about post-fusion events and RNA replication, such as nucleocapsid degradation. Recent studies have shown that ubiquitination plays an important role during this period.

Viruses hijack the process of ubiquitination in a proteasome-dependent manner to accelerate internalization, which can be explored by proteasome inhibitors, for example, MG132 and lactacystin (Schneider et al., 2021). Experiments with MG132 and lactacystin reduced the productive internalization of Japanese encephalitis virus (JEV) virions, but have no effect on viral attachment, penetration, or initial translation (Wang et al., 2016b; Figure 4). Therefore, the proteasome plays an important role in the internalization of JEV. However, cellular proteins degraded by proteasomes may not be conjugated with ubiquitin (Hicke, 2001). Ubiquitin-specific siRNAs were transfected to knock down the levels of free ubiquitin, which could lead to the same results (Wang et al., 2016b). Therefore, ubiquitination is evidenced to play important roles in the internalization of JEV. There may be specific E3 ligase interacting with JEV structural proteins and mediating the ubiquitination of the protein, and this topic deserves further study using proteomics screening methods or RNAi genome to identify such cofactors involved with JEV entry.

Studies have shown that the internalization process of the virus requires proteasome activity, but the release of viral genome from capsid protein is not related to the proteasome (Mercer et al., 2012). Ubiquitin inhibitors, such as PYR41 (the specific irreversible inhibitor of the ubiquitin-activating enzyme E1), are used to explore the effect of non-degraded ubiquitination in the process of uncoating. The uncoating of DENV genome is inhibited through the UBEI41 (PYR41) inhibitor targeting UBA1 in A549 cells (Byk et al., 2016; Figure 4), which result in the DENV inactivated virus RNA trapped in the endosome or nucleocapsid and DENV ultimately failed to complete its life cycle (Byk et al., 2016). During the yellow fever virus (YFV) infection, the E1 activity inhibitor PYR41 treatment also confirmed that ubiquitination is necessary for uncoating of YFV genome (Ramanathan et al., 2020). These studies substantiated that a non-degradable ubiquitination step is required during the uncoating process of DENV and YFV. Moreover, the proteasome mentioned above in the internalization process can degrade capsid protein for viral internalization (Byk et al., 2016).

In summary, the above studies not only clarified that ubiquitination is essential for virus internalization, but also revealed the exact stages (from fusion to pre-translation) involved in ubiquitination. However, specific ubiquitination-related factors and the substrate of ubiquitination are not known yet and need to be further studied. Recently, many potential E1 enzyme inhibitors have been developed, while clinical trials have not been carried out (Huang and Dixit, 2016).

### Ubiquitination Involved With Flaviviridae Virus Replication

After the viruses entering cells and the nucleocapsid uncoating, the ribosome recognizes the released positive-sense RNA, then the translation begins on the rough endoplasmic reticulum (ER) to produce a single polyprotein. Viral and cellular proteases catalyze the co-translation and the cleavage of viral polyprotein into structural proteins and non-structural proteins, and almost all structural proteins and non-structural proteins can attach with the intracellular membrane of the host (Rodenhuis-Zybert et al., 2010). The viral RNA is synthesized in the replication complexes, which composed of nonstructural proteins NS3, NS4A, NS4B, NS5 of *Flavivirus* (or NS5A and NS5B of other genera), viral RNA, and other cellular components (Moradpour et al., 2003; Risager et al., 2013). Host cells utilize ubiquitination mechanisms to interfere with virus’s replication, and viruses have evolved some strategies to usurp ubiquitination to climate their replication or escape immunity.

#### The Ubiquitination of NS3

There is much evidence that the triple motif (TRIM) family proteins play an important role in inhibiting virus replication (van Tol et al., 2017). The TRIM protein family is a group of E3 ligases, and many its members either indirectly act as components of innate immune signaling to limit virus replication or directly target viral proteins for degradation (Hage and Rajsbaum, 2019; Giraldo et al., 2020a).

**Figure 5:**
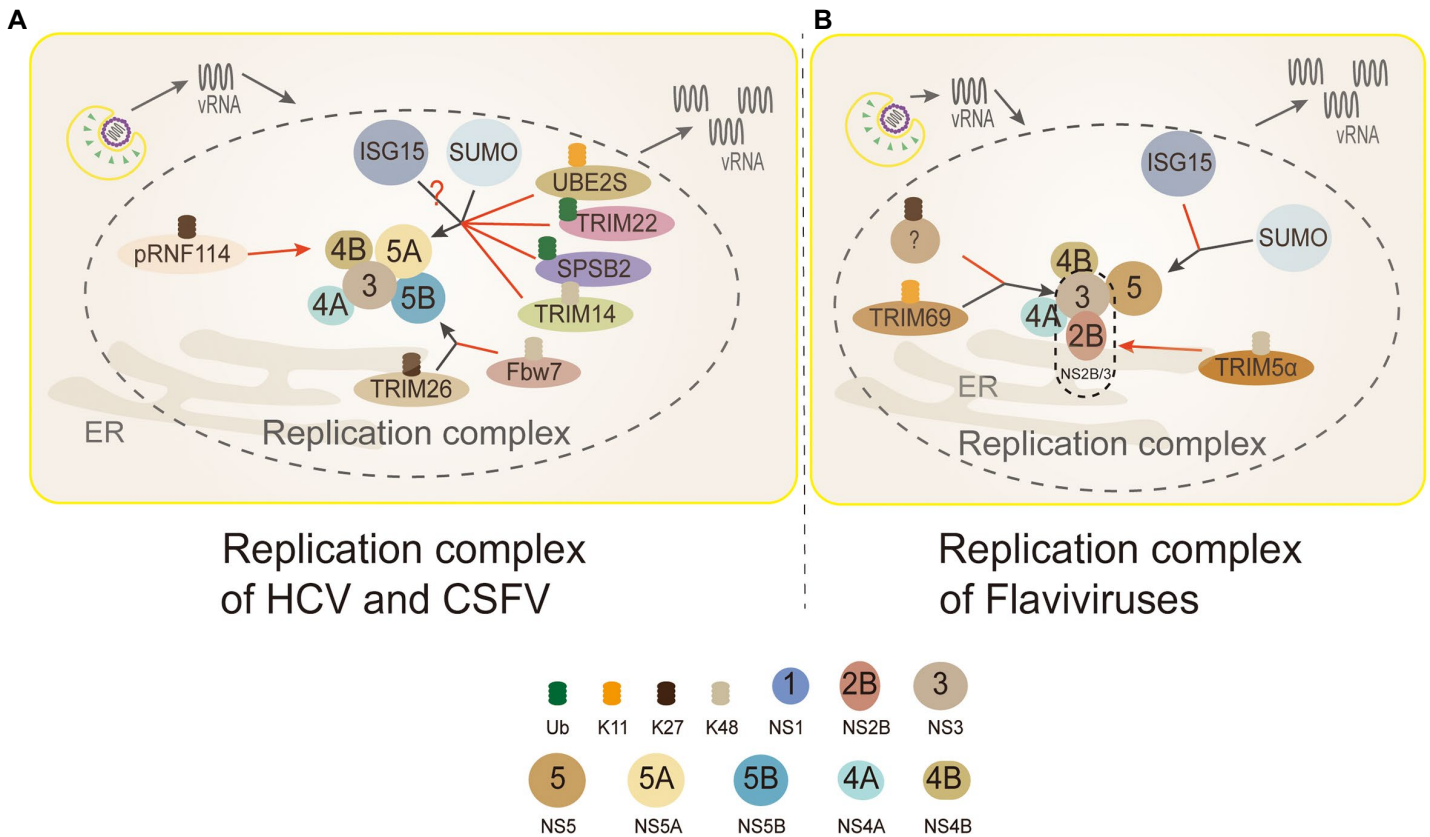
Ubiquitin in the replication complex of Flaviviridae. 3 (NS3): TRIM69 ubiquitinates virus NS3 protein in the K11-linked way, causing NS3 degradation to inhibit DENV replication. TRIM5α inhibits replication and promotes the ubiquitination of its K48-linked and proteasome degradation by binding to Flaviviruses virus protease NS2B/3. But, NS3 is modified by the K27-linked polyubiquitin to promote virus replication. 4B (NS4B): PRNF114 mediates the polyubiquitin of K27-linked NS4B, which degrades NS4B, through the proteasome pathway and then inhibits CSFV replication. 5A (NS5A): TRIM22 and SPSB2 directly target NS5A, and degrade it through ubiquitination to inhibit HCV replication. UBE2S and TRIM14 degrade NS5A protein through Lys11-linked and Lys48-linked proteasome-dependent pathways. SUMOylation improves the stability of NS5A protein by inhibiting ubiquitination, thus promoting the interaction between NS5A and NS5B protein. NS5A is the active target of ISGylation, but the roles of ISGylation remain obscure. 5B (NS5B): TRIM26-mediated K27-linked ubiquitination of NS5B at residue K51 enhances the interaction between NS5B and NS5A, thereby promoting the replication of the HCV. Fbw7 can interact with NS5B and degrade NS5B by K48-linked ubiquitination. **(A)** Replication complex of HCV and CSFV: PRNF114 mediates the polyubiquitin of K27-linked NS4B, which degrades NS4B through the proteasome pathway and then inhibits CSFV replication; TRIM22 and SPSB2 directly target NS5A, and degrade it through ubiquitination to inhibit HCV replication; UBE2S and TRIM14 degrade NS5A protein through Lys11-linked and Lys48-linked proteasome-dependent pathways. SUMOylation improves the stability of NS5A protein by inhibiting ubiquitination to promote the interaction between NS5A and NS5B protein; NS5A is the active target of ISGylation, however, whether the role of ISGylation is to promote or inhibit is controversial; TRIM26-mediated K27-linked ubiquitination of NS5B at residue K51 enhances the interaction between NS5B and NS5A to facilitate HCV replication; Fbw7 can interact with NS5B and degrade NS5B by K48-linked ubiquitination. Black arrows, promoting replication complexes; red arrows, inhibiting replication complexes. **(B)** Replication complex of Flaviviruses: TRIM69 ubiquitinates virus NS3 protein in the K11-linked way, causing NS3 degradation to inhibit DENV replication; the other studies show that NS3 is modified only by the K27-linked polyubiquitin to facilitate virus replication, and which linkages are needed to be discussed further; TRIM5α inhibits virus replication and promotes the ubiquitination of its K48-linked and proteasome degradation by binding to NS2B/3 (Flaviviruses protease); interferon stimulating gene 15 (ISG15) and sumo contribute to the stabilization of NS5 and promote virus replication.

There are some TRIM family proteins, such as TRIM69, TRIM5α, ubiquitinating key viral replication NS3 protein (NS2B/3) for degradation (Tay et al., 2015). DENV NS3 protein, a multifunctional protein with both serine protease and NTPase/helicase activity, is a key protein for DENV replication (Wang et al., 2009). TRIM69 was confirmed to ubiquitinate virus protein NS3 of DENV at residue Lys104 with a K11-linked way in 293T cells, which caused NS3 degradation (Wang K. et al., 2018). Yet, there is a study that suggests NS3 is exclusively modified by the K27-linked polyubiquitin to promote virus replication in HEK 293T cells (Liu et al., 2017; Figure 5). It’s obviously divergence between the two results (linkage K11 or K27) of NS3. Thus, more experiments are urgent needed to tackle the detailed form of ubiquitination on NS3.

TRIM5α, one of the most characteristic primate TRIM proteins, is a highly specific cellular antiviral restriction factor. In the past, it was thought to restrict retroviruses only through interactions with capsid lattice structures, which accelerating the uncoating of viral nucleic acids and preventing reverse transcription (Stremlau et al., 2006; Ganser-Pornillos et al., 2011). TRIM5α inhibits virus replication and promotes the ubiquitination of its K48-linked and proteasome degradation by binding to Flaviviruses protease NS2B/3 in HEK293 cells (Chiramel et al., 2019; Figure 5). NS3 can form a protease complex with NS2B (NS2B/3) to cleave *de novo* translated polyproteins and many other proteins (Assenberg et al., 2009; Tay et al., 2015). Hence, it is an indispensable protein for Flaviviruses translation and replication (Assenberg et al., 2009; Tay et al., 2015).

#### The Ubiquitination of NS5A

As an indispensable role in consisting replication complex, HCV NS5A is also a new ubiquitinated protein (Wang et al., 2016a, 2019; Yang et al., 2016; Pham et al., 2019). During HCV infection, the expression level Ubiquitin-conjugating enzyme E2S (UBE2S) and Lys11 chains were suppressed significantly, which made HCV-infected cells DNA damage to promote its replication (Pham et al., 2019). The results revealed that K11-linked ubiquitination participated in the proteasome-dependent degradation of HCV NS5A to inhibit the replication of HCV (Figure 5).

The inhibitory effect of many members of the TRIM family on viral replication has been verified on many studies of HCV (Wang et al., 2016a; Yang et al., 2016). TRIM14 and TRIM22, act as E3 ligases, directly target and degrade NS5A through ubiquitination to inhibit HCV replication (Wang et al., 2016a; Yang et al., 2016). Most TRIM family members correlate with the innate immune response, acting as interferon stimulating genes (ISGs) to inhibit the virus replication (Nisole et al., 2005). TRIM22 and TRIM14 are ISGs regulated by IFN (Yang et al., 2016). TRIM22 specifically binds, ubiquitinates, and degrades NS5A in a proteasome-dependent manner (Yang et al., 2016; Figure 5). TRIM14 interacts with NS5A and degrades NS5A in K48-linked way, which is mainly mediated by the SPRY domain of TRIM14 (Wang et al., 2016a; Figure 5). It appears that in the case of interferon resistance, TRIM22 and TRIM14 would provide a new direction for the treatment of patients with HCV. What needs to be further identified is the lysines of NS5A and the linkages ubiquitinated by TRIM22. Additionally, autophagy and ubiquitin proteasome-dependent degradation are two main ways for the host to degrade proteins, and the crosstalk between ubiquitin and autophagy has been uncovered. K48-linked ubiquitination of cGAS is a recognition signal for p62-depdendent selective autophagic degradation, and DNA sensor cGAS is closely related to interferon production (Chen M. et al., 2016; Hopfner and Hornung, 2020). TRIM14 can recruits USP14 to cleave K48-linked ubiquitin chains of cGAS, thereby inhibiting p62-cGAS interaction and cGAS autophagy degradation (Chen M. et al., 2016).

SPRY domain- and SOCS box-containing protein 2 (SPSB2), the adaptor protein of ECS (Elongin B/C-Cullin 5-SOCS box protein) E3 ligase complex, can recruit target protein to ubiquitinate and degrade them (Kuang et al., 2010). SPSB2 indirectly targets NS5A in SoCS box-dependent manner to promote NS5A ubiquitination and degradation in proteasome-dependent manner, and significantly inhibits HCV replication (Wang et al., 2019; Figure 5).

To sum up, NS5A is degraded through ubiquitination proteasome system (UPS) possibly mediated by K11 and K48 polyubiquitin, but the linkages of polyubiquitin corresponding to the specific ligase need to be further studied.

#### The Ubiquitination of NS5B

Another essential protein associated with HCV replication is NS5B, which has the RNA-dependent RNA polymerase (RdRp) activity (Manns et al., 2017). F-box and WD repeat domain-containing 7(Fbw7) is a recognition component of E3 ligase SCFF^Fw7^ (SKP1-CUL1-F-box protein) complex (Welcker and Clurman, 2008). The study showed that Fbw7 gene knockdown could promote HCV replication, while exogenous expression of Fbw7 could strongly inhibit HCV replication in HEK293T cells (Chen J. et al., 2016). Further research showed that HCV NS5B was degraded through the UPS. Fbw7 can recognize a CPD motif of HCV NS5B to degradation mediated by K48-linked ubiquitination (Chen J. et al., 2016; Figure 5).

The TRIM family can not only inhibit the virus infection, but some reports have shown that Flaviviridae viruses utilize the ubiquitination of TRIM family to promote their replication. TRIM26-mediated K27-linked ubiquitination of NS5B at residue K51 enhances the interaction between NS5B and NS5A in Huh7 cells, thus promoting the replication of HCV (Liang et al., 2021; Figure 5). The role of TRIM26 in HCV replication seems to be virus-specific, because it does not participate in the life cycle of other closely related Flaviviruses, for example, DENV and ZIKV. TRIM26 may be a unique ubiquitination ligase applied by HCV to benefit its replication (Liang et al., 2021).

#### The Ubiquitination of Other Proteins of Flaviviridae

NS1 plays an important role in the replication process by interacting with other viral protein including NS4B (Youn et al., 2012). NS1 was degraded after ubiquitination at K189 residues, leading to the reduction of interaction between NS1 and NS4B in Huh7 cells (Giraldo et al., 2018). Sine this interaction may play a role in the formation of replication complex (RC), the DENV replication was inhibited in this research. However, it is not clear what is the precise ubiquitination process of NS1. Fortunately, the ubiquitination of NS4B has been discovered. Ring domain E3 ligase (RINGE3s) is a group of E3 ligases containing the ring finger domain. Porcine RING finger protein 114 (pRNF114), a member of RINGE3s, degraded NS4B mediated by the polyubiquitin of K27-linked proteasome pathway, which could lead to the interruption of classical swine fever virus (CSFV) replication (Zhang Y. et al., 2019; Figure 5).

Flaviviridae viruses also can counteract the cellular ubiquitin system. The E6-associated protein (E6AP) is a ubiquitin ligase that mediates the ubiquitination and proteasome degradation of HCV core protein (Shirakura et al., 2007). Through direct infection and ectopic expression of HCV core protein, consequently, HCV core protein made the host promoter hypermethylation, which inhibited the expression of E6AP in HepG2 cells (Kwak et al., 2016). Therefore, the results collaborated that HCV can counteract the host antiviral defense mechanism of E6AP to produce infectious viral particles (Kwak et al., 2016).

### Ubiquitination Involved With Flaviviridae Virus Assembly and Release

After the viral protein cleaved, the virions assembly were carries out on the ER membrane, which is very close to the viral RNA replication site, usually directly opposite to the hole squeezed out of the virus replication compartment (Welsch et al., 2009; Junjhon et al., 2014; Cortese et al., 2017). The process of the assembly was involved in the formation of the viral nucleocapsid and envelop (Bartenschlager et al., 2011). After assembly, the new *Flavivirus* immature particles containing prM suffered from furin-mediated cleavage along the secretory pathway in Golgi. E1 and E2 of *Hepacivirus* contain high-mannose and complex N-links glycans, suggesting that the modified glycans of the virus particles may occur in Golgi apparatus. Finally, the mature particles are released by exocytosis.

The assembly of capsid (C) protein and viral RNA (vRNA) are critical for completion of the life cycle of Flaviviridae (Tan et al., 2020). West Nile Virus (WNV) C protein can be degraded by ubiquitination through UPS (Ko et al., 2010). Makorin ring finger protein 1 (MKRN1), an E3 ubiquitin ligase, can ubiquitinate C protein in the proteasome. MKRN1-mediated ubiquitination and mutant degradation are significantly inhibited when the three lysine sites at positions 101, 103, 104 of WNV capsid protein are replaced by alanine (Ko et al., 2010), which illustrates that these sites are necessary for ubiquitination.

Viruses commonly acquire their envelopes by recruiting the host endosomal sorting complex required for transport (ESCRT) machinery at the plasma membrane (Votteler and Sundquist, 2013). Hepatocyte growth factor-regulated tyrosine kinase substrate (HRS), one of the complex components of ESCRT-0, is an important entry medium for HCV to ESCRT pathway (Barouch-Bentov et al., 2016). NS2 of HCV can be ubiquitinated in a K63-linked fashion by membrane-associated RING-CH 8 (MARCH8), an E3 ligase located on the endosome and plasma membrane (Samji et al., 2014), which mediates HRS binding and envelop assembly of HCV (Kumar et al., 2019). NS2 ubiquitination plays an essential role in HCV envelopment, and also has been verified in DENV and ZIKV (Kumar et al., 2019). MARCH8 maybe essential for the assembly of other Flaviviridae family members, which may become a potential antiviral host target.

After assembly completion, virions egress from the cells surface. Ubiquitin proteasome pathway (UPP) plays a critical role in regulating DENV egress from infected cells (Choy et al., 2015a). Lactacystin (β-lactone), a widely used proteasome inhibitor, can increase the expression of DENV E protein by a mean of dose-dependent at 24 hpi, but NS3 protein did not change (Choy et al., 2015b). The results of confocal microscopy also showed that more accumulations of E and prM structural proteins were strong co-localization with Golgi markers in β-lactone treated cells than DMSO treated at 24 hpi. Electron microscopy displayed that 50 nm virus particles were present in intracellular vacuoles in both β-lactide or DMSO treated cells, suggesting that DENV RNA could replicate and be translated and packaged with structural proteins to form mature particles (Choy et al., 2015b).

During the virus release, the Flaviviruses prM protein is cleavaged by furin-mediated way in Golgi apparatus. In trans Golgi apparatus, prM proteins cleaved by furin proteases release pr peptides from prM-E protein heterodimer complexes, and eventually form M/E protein heterodimer complex. A recent study observed that prM protein can be ubiquitinated and degraded *via* UPS during ZIKV infection, while ZIKV prM protein ubiquitination contributes to the release of viral proteins (Nambala et al., 2020).

## Ubiquitin-Like Modifications

A group of proteins that are similar to ubiquitin in sequence and three-dimensional structure is collectively called ubiquitin-like modifiers (UBLs), and most UBLs are conjugated to the target protein through ubiquitin-like enzyme E1, E2, E3 cascade reactions (Hochstrasser, 2009). UBLs, such as small ubiquitin-like modifier (SUMO) and interferon stimulating gene 15 (ISG15), play an important role in the infectious process of Flaviviridae virus (Chen et al., 2010, 2013; Lee et al., 2014; Su et al., 2016; Conde et al., 2020).

### SUMOylation

In recent years, attentions have been paid to the interaction between various viruses and SUMOylation (Boggio and Chiocca, 2006; Wimmer et al., 2012). SUMOylation is a modification that works by SUMO, an 11 kDa molecule, covalently binding to lysine residues of the target protein (Wilkinson and Henley, 2010). SUMOylation is carried out by an E1 activating enzyme, an E2 conjugating enzyme (Ubc9), and an E3 ligase (Meulmeester and Melchior, 2008).

Several studies have shown that SUMO strengthens the non-structural protein 5/5A stability to support Flaviviridae replication (Lee et al., 2014; Su et al., 2016; Conde et al., 2020). Moreover, the interaction between the CSFV Core protein and host SUMO pathway serves as an irreplaceable role in the life cycle of CSFV (Gladue et al., 2010).

SUMO E2 enzyme Ubc9 takes part in a key step of protein SUMOylation (Chiu et al., 2007). Through siRNAs knockdown of the Ubc9, the HCV RNA replication decreased significantly in HEK293T cells (Lee et al., 2014). Furthermore, the co-immunoprecipitation experiment showed that SUMO targets on NS5A to promote its stability and interaction with NS5B (Lee et al., 2014). The lysine residues K348 of NS5A, the target site of SUMO, were mutated to arginine (K348R) leading to significantly decreased SUMOylation. Moreover, mutant NS5A cannot interact with NS5B, which indicates that SUMOylation of NS5A plays an important role in the form of viral replication complex. In addition, compared with the wild-type NS5A mutant, SUMO-defective HCV replicon cells had a higher level of ubiquitination, indicating that SUMOylation keep the stability of NS5A by inhibiting ubiquitination (Lee et al., 2014; Figure 5). It is suggested that the SUMOylation of NS5A may provide a potential target for the treatment and intervention of HCV.

In the genus *Flavivirus*, SUMO has also been shown to play a key role in the stability of NS5 (Su et al., 2016; Zhu et al., 2019). Besides, NS5 not only participate in viral RNA replication, but also in innate immune antagonism (Wang B. et al., 2018). The level of SUMOylation was suppressed by the knockdown of Ubc9, which lead to the reduction of DENV replication (Su et al., 2016). Further experiments also showed that SUMOylation-defective NS5 destroyed DENV RNA replication and innate immune antagonism (Su et al., 2016). Multiple sequence alignments of 78 representative species of *Flaviviruses* revealed that most of them (72/78, 92.3%) had a hypothetical SUMO-interacting motif (SIM) in the N-terminal region of their NS5 (Zhu et al., 2019). It is suggested that the N-terminal SIM motif is necessary for the SUMOylation of NS5 protein (Su et al., 2016). Particularly, DENV NS5 needs a functional SIM to stabilize its biological function, and the NS5 SUMOylation may govern DENV replication by regulating protein turnover of the replication complex (Su et al., 2016). The similar effect of SUMOylation on NS5 in ZIKV has also been verified (Zhu et al., 2019).

2-D08 (2′,3′,4′-trihydroxyflavone), a SUMOylation inhibitor, can block the transfer of SUMO-1 from the E2 enzyme Ubc9 to the substrate (Kim et al., 2013). 2-D08 significantly reduced ZIKV replication and protected cells from cytopathic effects (Zhu et al., 2019). The inhibitory effect of SIM mutant ZIKV NS5 on interferon-β induced interferon-stimulated response element (ISRE)-dependent luciferase activity was significantly lower than that of wild type ZIKV NS5 (Zhu et al., 2019). Compared with wild type, the inhibitory effect of SIM mutant ZIKV NS5 on interferon-β induced ISRE-dependent luciferase activity was significantly lower than that of wild type ZIKV NS5 (Zhu et al., 2019). It is suggested that SUMOylation is essential for type I interferon signal transduction mediated by ZIKV NS5 (Zhu et al., 2019). In general, these findings may suggest that the SUMOylation of viral NS5 protein is an evolutionarily conservative post-translational modification process among *Flaviviruses*, which promotes the stability of NS5 protein to enhance viral replication and inhibit host antiviral response. Therefore, reducing the SUMOylation to disrupt NS5 protein stability may be a new potential target for drug design to resist *Flavivirus*.

### Interferon Stimulating Gene 15

ISG15 is found as the first ubiquitin-like protein modifier. ISG15 can bind to its cellular target through a series of enzymatic steps (a process known as ISGylation). Firstly, ISGylation involves E1 activating enzyme (Ube1L), then E2 conjugating enzyme (UbcH8), and finally E3 ligase (EFP, CEB1/Herc5 or HHARI; Zhao et al., 2004; Okumura et al., 2007). ISGylation works like the ubiquitin system and even overlaps with the ubiquitin system, it means that E2 (UbcH8) and E3 (Herc5) are shared by the two systems (Wong et al., 2006). ISG15, one of the most abundant proteins induced by virus infection or type I interferon (IFN-I), is an executor of host innate immune to inhibit the *Flaviviruses* (Hsiao et al., 2010; Dai et al., 2011; Kim et al., 2013; Hishiki et al., 2014; Singh et al., 2019; Li et al., 2020).

The overexpression of ISG15 in human medulloblastoma cells significantly reduced the cytopathic effect induced by JEV, inhibited the replication of JEV, and decreased the activation of ISRE (Hsiao et al., 2010). After silencing ISG15 with siRNAs, the viral load of DENV, WNV, and ZIKV was significantly higher than that in wild-typed cells (Dai et al., 2011; Hishiki et al., 2014; Singh et al., 2019). Recently, it has been reported that ISG15 inhibited CSFV replication by blocking autophagy (Li et al., 2020). The overexpression of Flag-ISG15 significantly inhibited CSFV replication, whereas the knockdown of ISG15 led to the abnormal CSFV proliferation (Li et al., 2020). In addition, up-regulated ISG15 can promote ISGylation and destroy the function of Beclin-1 (BECN1), leading to inhibition of autophagy that is necessary for CSFV replication (Li et al., 2020). There are many researches on the inhibitory effect of ISGylation on *Flaviviruses*, but the specific mechanism is rarely reported. Maybe targeting activation of ISG15 is a potential antiviral drug development strategy.

On the contrary, there are two different opinions on the role of ISGylation played in HCV replication (Broering et al., 2010; Chen et al., 2010, 2011; Kim and Yoo, 2010; Abe et al., 2020). Overexpression of ISG15 and ISG15-conjugation enzymes both can inhibit HCV replication, and NS5A is identified as the substrate of ISGylation (Kim and Yoo, 2010). There are five lysine residues (K309, K331, K349, K359, and K379) located in the C-terminal domain of HCV NS5A. The K309, K331, K349, K359, and K379 are conserved in different HCV isolates. Through the lysine residues replaced by arginine residues to knockdown the ISGylation, only the K379R mutant worked, whereas overexpression of ISGylation basic components did not revert this result (Kim and Yoo, 2010). It is suggested that the K379 of NS5A serves as the active site of ISGylation, which reduces the protein stability of NS5A and inhibits the HCV replication (Figure 5).

However, there are some reports that ISG15 promotes the production of HCV (Broering et al., 2010; Chen et al., 2010, 2011; Abe et al., 2020). In human and mouse cell lines, the level of HCV increased with the increase of ISG15 and ISGylation levels, vice versa (Broering et al., 2010; Chen et al., 2010). By co-immunoprecipitation assay, ISG15 interacts specifically with HCV NS5A protein and locates in domain I (Minami et al., 2017). Among the 14 Lys residues of NS5A (genotype 1b), 5 Lys residues (K44, K68, K166, K215, and K308) have the potential to accept ISGylation (Abe et al., 2020). Furthermore, the NS5A ISGylation of different genotypes of HCV was detected, and it was observed that NS5A of all HCV genotypes occurred the ISGylation of multiple Lys residues. HCV luciferase reporter replicon analysis showed that the K308 residue of NS5A was important for HCV (1b) replication. K308, one of the NS5A ISGylation-lysine residues, is located in the Cyclophilin A (CypA) binding region of NS5A, and CypA is the key host factor for HCV replication. Meanwhile, NS5A ISGylation enhanced the interaction between NS5A and CypA, indicating that NS5A ISGylation functions as a proviral factor, and promotes HCV replication by recruiting CypA (Abe et al., 2020; Figure 5). However, more investigations are needed to explore whether ISGylation is the only way to promote the HCV replication.

Generally speaking, overexpression of ISG15 showed anti-*Flavivirus* effects. However, it is not clear whether NS5A ISGylation plays the proviral or antiviral role in HCV replication. Accordingly, the triple therapy consisting of ISG15 knockout, interferon-α (IFN-α), and ribavirin completely inhibited HCV NS5A protein, equivalent inhibition rate to 99% of the HCV-RNA, whereas combining IFN-α and ribavirin had an inhibition rate of 75%. The combination of ISG15 gene knockout and IFN-α can enhance and prolong the expression of IFN-induced gene (Broering et al., 2010). Hence, treatment based on inhibition of ISG15 may provide a strategy for overcoming the non-response of interferon-α and ribavirin combination therapy in the future.

## Discussion and Outlook

This review highlights the roles of ubiquitination in Flaviviridae virus-host interactions. The Flaviviridae viruses exploit the ubiquitin and ubiquitin-like system at different stages of their life cycle, stressing the importance of this cellular mechanism for viral infection.

It is worth noting that the ubiquitination site of E protein is conserved in the Flaviviridae during the virus invasion (Dai et al., 2016), and it has achieved good results in the K63 neutralizing antibody test (Giraldo et al., 2020b), proposing it as the target of antiviral drugs against the Flaviviridae viruses. On the one hand, the detailed process of ubiquitination during the assembly and release stage are needed to be explored. On the other hand, the situation of the virus constantly evolved strategies against the host antiviral mechanism to survive should be alerted. There must be many ways that viruses seize the ubiquitination system to evade the cellular immunity have not yet been discovered. Prospective research should be focused on identifying the detailed step, linkage, and action site where ubiquitination occurs to pave ways for virus infection, and whether these processes are conserved in different viruses should be considered.

In recent years, many studies *in vitro* about modification of ubiquitin-related process to inhibit virus have been published. However, clinical trials have not been conducted in relation to ubiquitin. Direct-acting antivirals (DAAs) against viral NS3 protease, NS5A, and NS5B polymerase, are very effective in the treatment of patients with HCV has been reported widely (Bukh, 2016; Falade-Nwulia et al., 2017). The ubiquitination and ubiquitin-like modifications of NS3, NS5 (NS5A/NS5B) have been extensively reported above. It is inferred that inhibitor targeting ubiquitin site in NS3, NS5A and NS5B also may have similar effects. Hence, research in ubiquitination of viral proteins can provide enlightenment to find an effective treatment. Autophagy and ubiquitin-proteasome are two major pathways that mediate protein degradation. There are crosstalk between ubiquitin system and autophagy proved in many studies (Sparrer et al., 2017; Hopfner and Hornung, 2020), but there are few studies concerning this aspect in Flaviridae. Nevertheless, current works suggest a growing arena for therapeutic interventions against Flaviridae viruses.

## Author Contributions

DC conceived and designed the review. LL wrote the manuscript. BT raised issues and revised the manuscript. XF, QY, JC, and YZ gleaned the material. JF and LS designed and drew the figures. YW and LG sorted out the reference materials. ZZ coordinated the work and secured research funding. All authors contributed to the article and approved the submitted version.

## Funding

This research was funded by the China Agriculture (Beef Cattle/Yak) Research System (CARS-37) and the Sichuan Beef Cattle Innovation Team of National Modern Agricultural Industry Technology System (grant no. SCCXTD-2020-13).

## Conflict of Interest

The authors declare that the research was conducted in the absence of any commercial or financial relationships that could be construed as a potential conflict of interest.

## Publisher’s Note

All claims expressed in this article are solely those of the authors and do not necessarily represent those of their affiliated organizations, or those of the publisher, the editors and the reviewers. Any product that may be evaluated in this article, or claim that may be made by its manufacturer, is not guaranteed or endorsed by the publisher.
